# Clinicopathologic predictors of central lymph node metastases in clinical node-negative papillary thyroid microcarcinoma: a systematic review and meta-analysis

**DOI:** 10.1186/s12957-022-02573-7

**Published:** 2022-04-01

**Authors:** Xingzhu Wen, Qianmei Jin, Xiaoxia Cen, Ming Qiu, Zhihong Wu

**Affiliations:** 1 Department of General Surgery, 72nd Group Army Hospital, Huzhou University, Huzhou, Zhejiang, 313000 China; 2 Department of Rheumatology and Immunology, Changzheng Hospital affiliated to the Second Military Medical University, Shanghai, 200003 China; 3 Department of General Surgery, Changzheng Hospital affiliated to the Second Military Medical University, Shanghai, 200003 China

**Keywords:** Papillary thyroid microcarcinoma, Central lymph node metastases, Risk factor, Meta-analysis

## Abstract

**Background:**

The presence of central lymph node metastases (CLNM) has been suggested as a risk factor for poorer prognosis and recurrence in papillary thyroid microcarcinoma (PTMC). However, the clinicopathologic factors for CLNM in clinical node-negative (CN0) PTMC were not well defined. This study aimed to perform a systematic review and meta-analysis to investigate the significant clinicopathologic predictors of CLNM in CN0 PTMC.

**Methods:**

A systematic literature search was performed in PubMed, Embase, Cochrane Library, and Web of Science. Case-control studies on the association of clinicopathologic risk factors with CLNM in CN0 PTMC were included.

**Results:**

Thirteen eligible studies involving 6068 patients with CN0 PTMC were included. From the pooled analyses, male (odds ratio [OR]: 2.07, 95% *CI*: 1.49–2.87, *P* < 0.001), multifocality (*OR*: 1.88, 95% *CI*: 1.54–2.29, *P* < 0.001), tumor size > 5 mm (*OR*: 1.84, 95% *CI*: 1.55–2.18, *P* < 0.001), and extrathyroidal extension (*OR*: 1.96, 95% *CI*: 1.30–2.95, *P* = 0.001) are significantly associated with increased risk of CLNM in CN0 PTMC. A sample size with a cutoff point of 200 was identified as the source of heterogeneity for sex according to meta-regression (*t* = 3.18, *P* = 0.033). Then, the subgroup analysis of male was performed, which illustrated that male increased the risk of CLNM in the small sample group (SG) and the large sample group (LG) by 6.11-folds and 2.01-folds, respectively (SG: *OR*, 6.11, 95% *CI*, 3.16–11.81, *P* < 0.001; LG: *OR*, 2.01, 95% *CI*, 1.65–2.46, *P* < 0.001).

**Conclusions:**

Male, multifocality, tumor size > 5 mm, and extrathyroidal extension may be reliable clinical predictors of CLNM in CN0 PTMC. Moreover, prophylactic central lymph node dissection should be considered in surgical decision-making for CN0 PTMC patients, who are male, multifocal, with tumor size > 5 mm, and with extrathyroidal extension.

**Trial registration:**

CRD42021242211 (PROSPERO)

**Supplementary Information:**

The online version contains supplementary material available at 10.1186/s12957-022-02573-7.

## Introduction

Papillary thyroid carcinoma (PTC) has received much attention over the past decade due to its rapid global increasing incidence, with an incidence of 4.3/100000 to 143.3/100000 in women in various populations worldwide [1, 2]. Owing to the improved ability to detect small tumors on ultrasound imaging, most PTCs are initially diagnosed as papillary thyroid microcarcinomas (PTMCs), defined as the largest diameter of a PTC tumor ≤ 1 cm [3, 4]. Recently, total thyroidectomy or lobectomy plus ipsilateral prophylactic central lymph node dissection (CLND) are important recommended surgical approaches in the management of PTC [5–7]. However, prophylactic CLND in clinical node-negative (CN0) PTMC remains controversial due to the relatively indolent progression and potential risks of neurological and parathyroid dysfunction [8]. The 2015 American Thyroid Association guidelines strongly recommended that thyroidectomy without prophylactic CLND is appropriate for noninvasive CN0 PTMC [5]. Nevertheless, the previous studies revealed that the proportion of central lymph node metastases (CLNM) of CN0 unilateral PTMC was even higher than 40% [9–12]. In previous studies, the presence of CLNM was a significant factor related to recurrence-free survival in PTMC [13]. In addition, a meta-analysis including 25 studies revealed that prophylactic CLND significantly reduced the risk of central neck recurrence [14]. All of the above suggests that the incidence of CLNM in CN0 PTMC was not low enough to ignore the necessity of prophylactic CLND. Therefore, preoperative and intraoperative prediction of CLNM status in CN0 PTMC plays an important reference role in surgical decision-making. To explore the association between CLNM and clinicopathologic risk factors in CN0 PTMC, many case-control studies were completed [9–12, 15–23]. Clinicopathologic factors, such as sex, age, tumor size, multifocality, extrathyroidal extension (ETE), bilateral, capsule involvement, etc., were conferred candidate predictors for CLNM in CN0 PTMC [9–12, 15–23]. However, the existing single-center retrospective studies had the limitations of small sample size and inconsistent definitions of risk factors that might draw unreliable conclusions with considerable biases. Therefore, comprehensive systematic research with more objectivity and a larger sample size to investigate the predictors of CLNM in CN0 PTMC is needed. The purpose of this study is to perform a systematic review and meta-analysis to evaluate the preoperative and intraoperative clinicopathologic predictors of CLNM in CN0 PTMC.

## Materials and methods

This systematic review an meta-analysis were conducted according to the guidelines proposed by the Preferred Reporting Items for Systematic Reviews and Meta-analyses (PRISMA) [24]. Online registration has been accepted by PROSPERO (CRD42021242211).

### Search strategy

A literature search was conducted from PubMed, Embase, Cochrane Library, and Web of Science for articles published until February 11, 2022. Two independent authors (XZ Wen and QM Jin) identified articles with a combination of the following terms: “Papillary Thyroid Microcarcinoma,” “Lymphatic Metastasis,” “Risk factors,” and “Case-Control Studies” with language restriction “English.” All subject words and related random words were used for the search. The detailed search strategies were documented in Supplementary file 1.

### Study selection

All articles obtained from the search were screened according to the inclusion criteria: (1) original English articles; (2) CN0 PTMC patients who received total thyroidectomy or lobectomy and CLND as the primary surgical, and the CN0 was defined as no preoperative clinical evidence of lymph node involvement by ultrasonography; (3) study of the association between CLNM and relevant clinicopathologic and/or ultrasonic risk factors; (4) multivariate regression analysis was used to return at least one statistically significant risk factor expressed by odds ratio (OR) and 95% confidence interval (CI); and (5) case-control study. The selection process was screened by titles and abstracts initially to exclude the duplicates, conference abstracts, reviewers, meta-analysis, and non-English articles, and then the full text of the remaining studies was downloaded and assessed independently by two investigators (XZ Wen and QM Jin) based on the above inclusion criteria. A third investigator (XX Cen) was introduced to resolve the disagreements.

### Data extraction

The following data were extracted by two investigators (XZ Wen and QM Jin) independently in standardized forms: first author, the published year, country, sample size, number and percentage of males, mean age at diagnosis, number and percentage of CLNM patients, type of surgery, tumor size, multifocality, extrathyroidal extension, and bilateral and capsule involvement. Disagreements were resolved through consultation with the third investigator (XX Cen).

### Quality assessment

Each study’s risk of bias was assessed based on the Newcastle-Ottawa scale (NOS), which consists of three columns: selection, comparability, and exposure. A score of more than 6 stars was considered to be of high quality. Two investigators (XZ Wen and QM Jin) performed quality assessment independently, and the disagreements were resolved by discussing it with the third investigator (XX Cen).

### Statistical analysis

The pooled ORs and 95% CIs of the significant risk factors for CLNM were calculated. The fixed-effect model or the random-effect model was chosen according to the heterogeneity. *Cochran’s chi-squared* and *Higgins’ I*^2^ statistic were applied to evaluate the heterogeneity, with considerable heterogeneity defined as *P* < 0.10 or *I*^2^ > 50%. When moderate or severe heterogeneity existed, the sensitivity analysis used metainf meta-based influence analysis tool, and meta-regression was performed to explore the sources of considerable heterogeneity. The risk of publication bias was analyzed using *Egger’s test* and *funnel plot*. When the publication bias was presented, the trim and filling analysis was performed to calibrate the bias. All statistical analyses were processed using ReVman software 5.3 (Cochrane Collaboration, Oxford, UK) and Stata software 16.0 (Stata Corporation, College Station, TX, USA). All images were created with Adobe Photoshop CS6 13.0 (Adobe Corporation, San Jose, USA) for Windows.

## Results

### Description of literature search

**Fig. 1:**
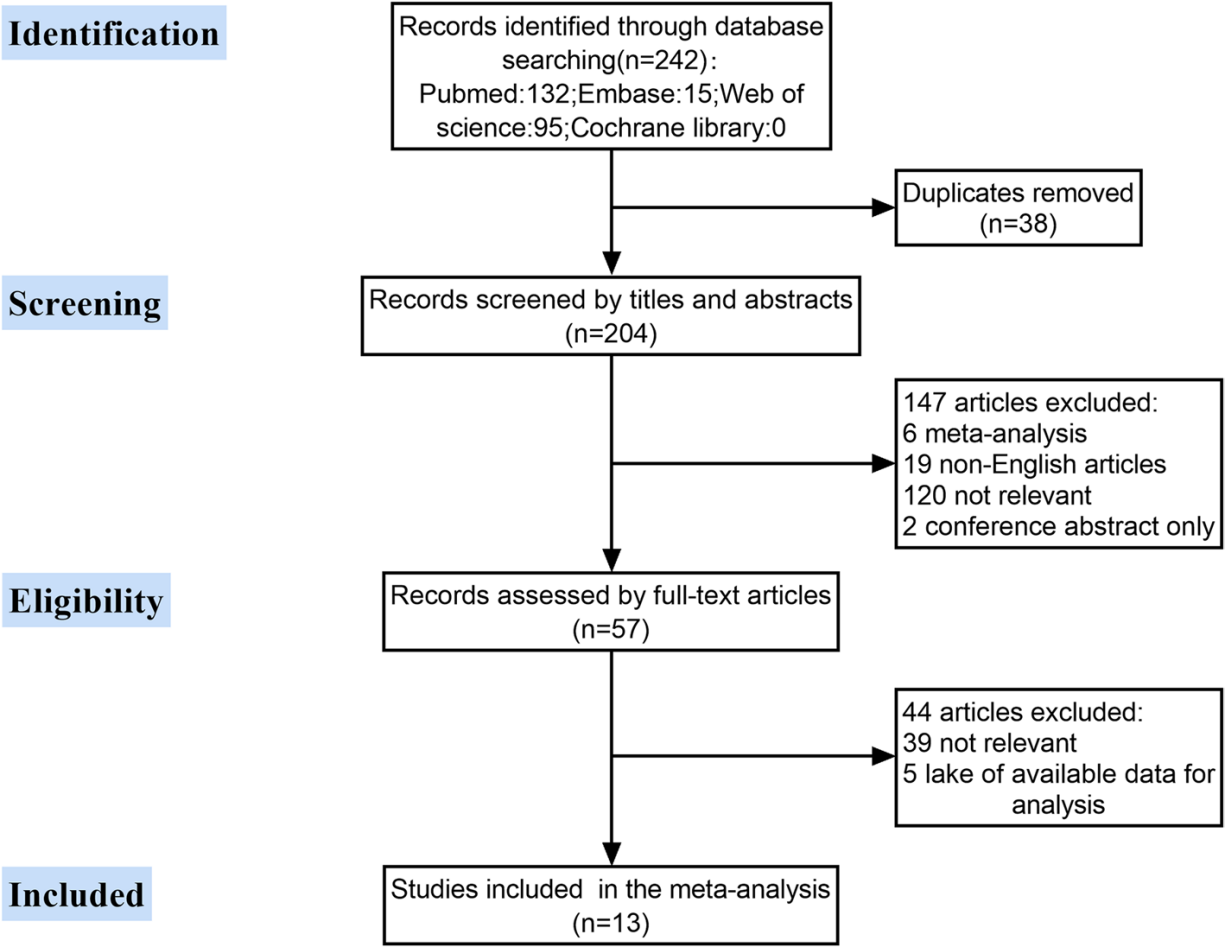
Flow diagram of literature search for this meta-analysis

As shown in Fig. 1, we identified a total of 242 studies through the database searching, then 38 duplicate studies, and a further 147 studies were excluded after titles and abstracts assessment. Next, a further 44 studies were also excluded by screening the full text of the remaining 57 articles. Finally, 13 case-control studies incorporating 6068 patients were included in this meta-analysis.

### Characteristics of the included studies

The characteristics of included studies are shown in Table 1, which lists author, published year, country, sample size, mean age, male, surgery type, CLNM, and significant risk factors. There were eight, five, ten, six, six, two, two, two, and one studies that investigated the sex, age, tumor size, tumor location, multifocality, extrathyroidal extension, bilateral, capsule involvement, tumor location, and Hashimoto’s thyroiditis (HT), respectively. The risk factors investigated in at least 3 studies were included in this meta-analysis. However, age was excluded due to the inconsistent definition.

**Table 1:** Characteristics of included studies

First author, year (references)	Country	Sample size	Mean age, y	Male (%)	Surgery type	CLNM (%)	Risk factors
Gui, 2018 [14]	China	541	47.2	128 (23.7)	TT/LT + CLND	148 (27.4)	S, A, TS, M, ETE
Zhou, 2012 [9]	China	122	48.0	60 (49.2)	TT + CLND	60 (49.2)	S, A, TS
Yuan, 2017 [10]	China	295	43.0	89 (30.2)	TT/LT + CLND	125 (42.4)	A, M
Kim, 2012 [15]	Korea	160	47.3	19 (11.9)	TT + B-CLND	61 (38.1)	S, TS
Park, 2014 [16]	Korea	193	49.2	32 (16.6)	TT/LT + CLND	63 (32.6)	TS, M
Zhang, 2015 [11]	China	178	46.0	37 (20.8)	TT/LT + CLND	73 (41.0)	S, TS, ETE
Xu, 2014 [17]	China	402	45.4	79 (19.7)	TT/LT + CLND	156 (38.8)	TS, B, CI
So, 2010 [18]	Korea	551	50.2	111 (20.1)	TT + B-CLND	202 (36.7)	S, M, ETE
Caliskan, M 2012 [19]	Korea	842	46.3	73 (8.7)	TT/LT + CLND	218 (25.9)	TS, ETE

*CLNM* central lymph node metastases, *TT* total thyroidectomy, *LT* lobectomy, *CLND* central lymph node dissection, *B-CLND* bilateral-central lymph node dissection, *S* sex, *A* age, *TS* tumor size, *M* multifocality, *ETE* extrathyroidal extension, *B* bilateral, *CI* capsule involvement

### Assessment of study quality

According to the NOS scale, the risks of bias of the included studies were evaluated. Among the included studies, 8 studies scored 7 stars and the other 5 studies scored 8 stars. All included studies had more than 6 stars, which were considered of high quality. The specific scores are shown in Table 2.

**Table 2:** Quality assessment of Newcastle-Ottawa scale

First author, year (references)	Selection	Comparability	Exposure	Score
Gui, CY 2018 [15]	☆☆☆	☆	☆☆☆	7 stars
Zhou, YL 2012 [9]	☆☆☆	☆☆	☆☆☆	8 stars
Yuan, J 2017 [10]	☆☆☆	☆☆	☆☆☆	8 stars
Kim, BY 2012 [16]	☆☆☆	☆☆	☆☆☆	8 stars
Park, JP 2014 [17]	☆☆☆	☆	☆☆☆	7 stars
Zhang, LY 2015 [11]	☆☆☆	☆	☆☆☆	7 stars
Xu, D 2014 [18]	☆☆☆	☆	☆☆☆	7 stars
So, YK 2010 [19]	☆☆☆	☆☆	☆☆☆	8 stars
Caliskan, M 2012 [20]	☆☆☆	☆	☆☆☆	7 stars
Zhang, Q 2019 [21]	☆☆☆	☆	☆☆☆	7 stars
Feng, JW 2020 [22]	☆☆☆	☆	☆☆☆	7 stars
Zhang, C 2020 [23]	☆☆☆	☆☆	☆☆☆	8 stars
Liu, C 2021 [12]	☆☆☆	☆	☆☆☆	7 stars

### The effect of sex on CLNM in CN0 PTMC

**Fig. 2:**
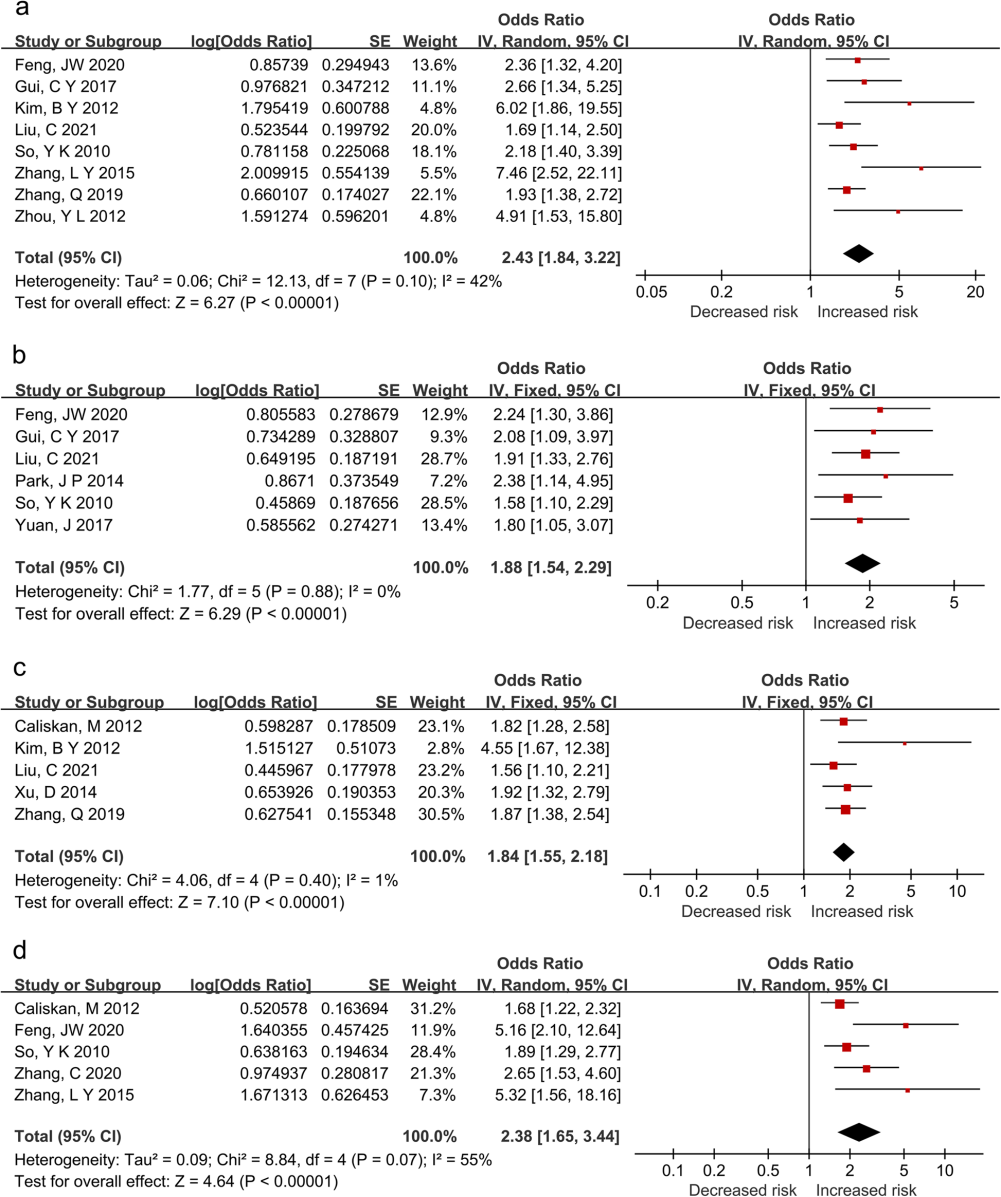
Forest plots for the effects of clinicopathologic risk factors on CLNM in CN0 PTMC. **a** Male. **b** Multifocality. **c** Tumor size > 5mm. **d** ETE

There were 8 studies that revealed sex was an independent risk of CLNM in CN0 PTMC. Due to the moderate heterogeneity (*P* = 0.096 < 0.1; *I*^2^ = 42.3% < 50%), a random-effects model was applied to the pooled analysis. As shown in Fig. 2A, the male was increasing 2.43-folds CLNM risk to female in CN0 PTMC patients (*OR*, 2.43; 95% *CI*, 2.43–3.22.; *P* < 0.001).

### The effect of multifocality on CLNM in CN0 PTMC

Multifocality was investigated as a significant risk of CLNM in CN0 PTMC in 6 studies. Due to the low heterogeneity (*P* = 0.88 > 0.1; *I*^2^ = 0% < 50%), a fixed-effects model was applied in the pooled analysis. As shown in Fig. 2B, the risk of CLNM in CN0 PTMC was significantly higher in patients with multifocality (*OR*, 1.88; 95% *CI*, 1.54–2.29; *P* < 0.001).

### The effect of tumor size on CLNM in CN0 PTMC

Tumor size was identified as a significant independent risk factor for CLNM in CN0 PTMC in 10 studies. However, only 5 studies that investigated tumor size > 5 mm as an independent risk factor were included in the final pooled analysis owing to the consistent definition. Due to the low heterogeneity (*P* = 0.40 > 0.1; *I*^2^ = 1.5% < 50%), a fixed-effects model was applied. As shown in Fig. 2C, the risk of CLNM in CN0 PTMC was significantly higher in patients, whose tumor size was > 5 mm (*OR*, 1.84; 95% *CI*, 1.55–2.18, *P* < 0.001).

### The effect of ETE on CLNM in CN0 PTMC

ETE was identified as a significant independent risk factor for CLNM in CN0 PTMC in 6 studies. Unfortunately, one study was excluded due to the definition of gross ETE, which was defined as macroscopic tumor extension outside the thyroid gland based on physical examination. A random-effects model was applied in the final pooled analysis due to the considerable moderate heterogeneity (*P* = 0.07 < 0.1; *I*^2^ = 54.7% > 50%). As shown in Fig. 2D, ETE significantly increased the risk of CLNM in CN0 PTMC patients by 2.38-folds (*OR*, 2.38; 95% *CI*, 1.65–3.44; *P* < 0.001).

### Sensitivity analysis

The sensitivity analysis was initially performed to assess the stability of the above pooled analyses. Overall, the leave-one-out analysis revealed that the pooled analysis results and the heterogeneities of multifocality and tumor size did not alter significantly when single studies were excluded. However, four studies and one study were identified for candidate sources of moderate heterogeneities in sex and ETE, respectively.

### Publication bias

**Fig. 3:**
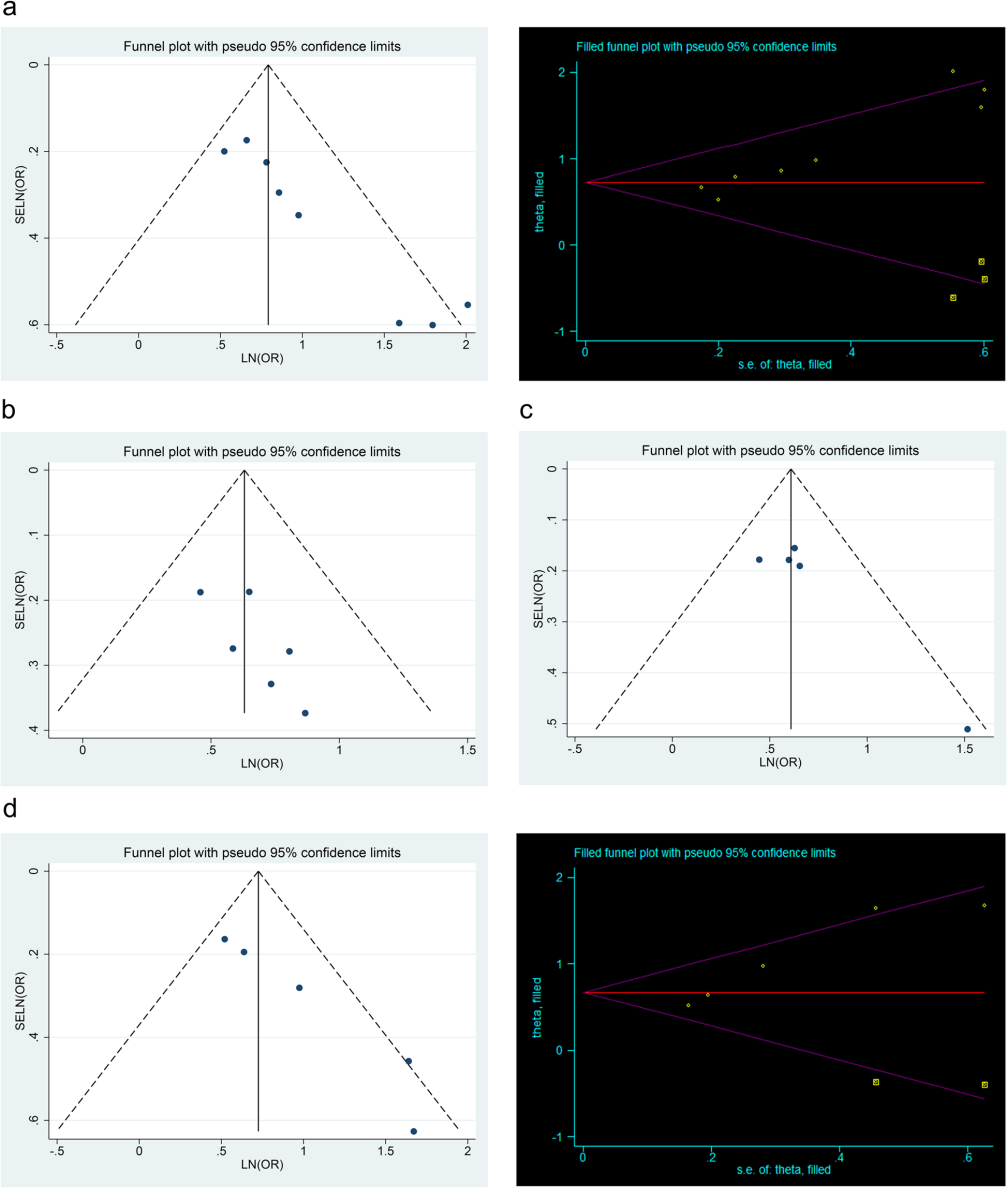
Publication bias of the clinicopathologic risk factors of CLNM in CN0 PTMC. **a** Funnel plots (left) and trim plots (right) of male. **b** Funnel plots of multifocality. **c** Funnel plots of tumor size > 5mm. **d** Funnel plots (left) and trim plots (right) of ETE

The publication bias was evaluated by the funnel plot and the Egger’s test (Fig. 3). Unfortunately, significant publication biases were found in sex (*P* < 0.001) and ETE (*P* = 0.006). We then applied the trim and filling analysis to calibrate the biases of sex and ETE by filling three and two studies respectively, which eventually conducted the calibrated values of ORs of male and ETE were 2.07 and 1.96 (male: *OR*, 2.07, 95% *CI*, 1.49–2.87; *P* < 0.001; ETE: *OR*, 1.96, 95% *CI*, 1.30–2.95; *P* = 0.001).

### Heterogeneity analysis of sex and ETE

Based on the sensitivity analyses, country, type of surgery, and sample size were identified as suspicious sources of heterogeneity for sex and ETE by carefully reviewing the recorded variables of the included studies. Then the meta-regression was performed. Fortunately, only the sample size with a cutoff point of 200 was a significant variable in sex (*t* = 3.18, *P* = 0.033), suggesting that the sample size might be the true source of heterogeneity for sex. However, no significant variables were found in the meta-regression of ETE.

### Subgroup meta-analysis of sex based on sample size

**Fig. 4:**
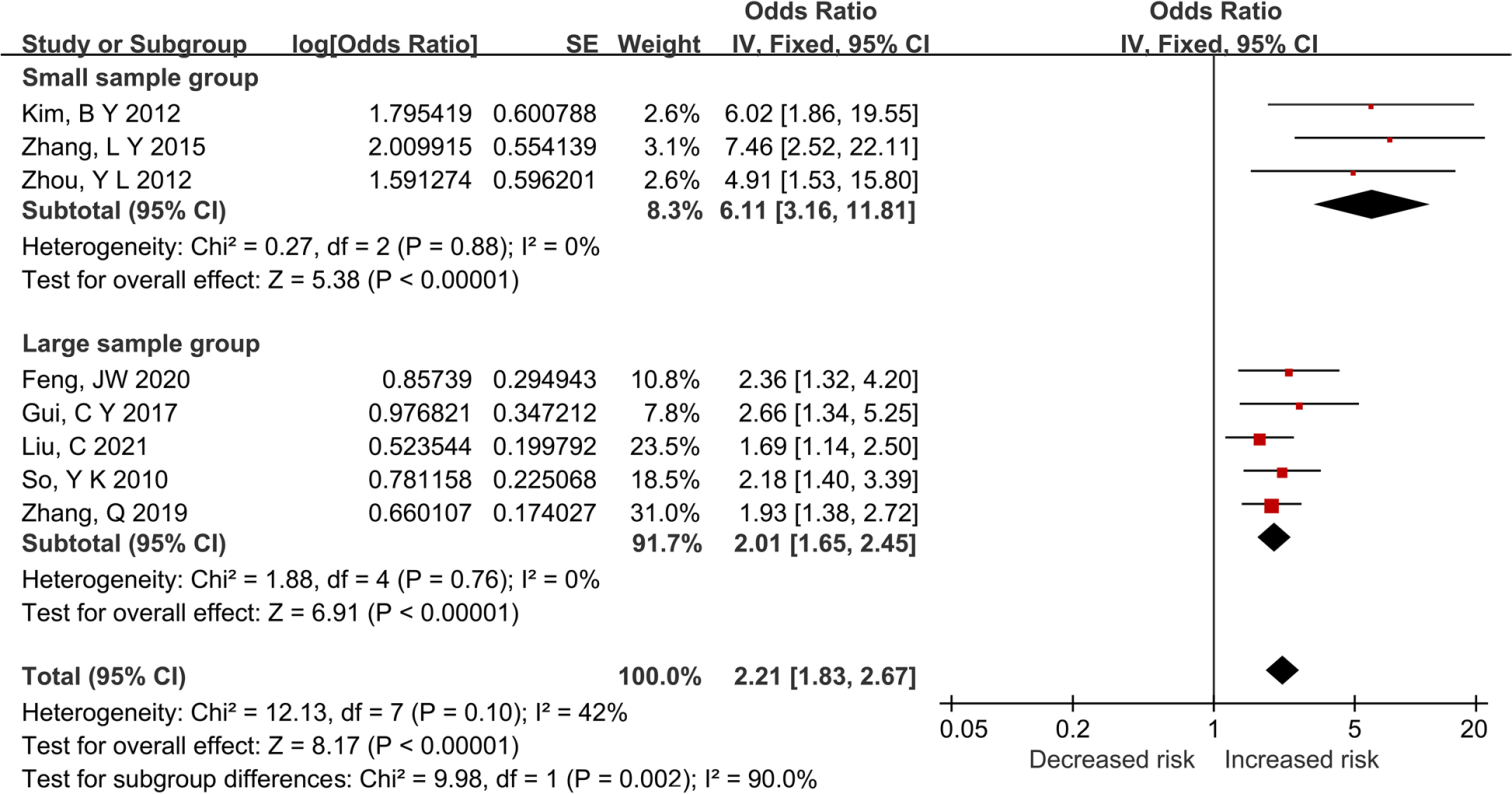
Forest plots of subgroup analysis for the effect of male on CLNM in CN0 PTMC based on sample size

According to the sample size, the 8 included studies that investigated sex were divided into 2 subgroups: small sample group (SG) with a size < 200 (*n* = 3) and large sample group (LG) with a size > = 200 (*n* = 5). Fixed-effect models were both applied in 2 subgroups due to the absence of heterogeneities (SG, *P* = 0.88 > 0.1; *I*^2^ =0% < 50%; LG, *P* = 0.76 > 0.1; *I*^2^ = 0% < 50%). Moreover, significant difference in heterogeneity was observed between subgroups (*P* = 0.002). As shown in Fig. 4, the male was increasing 6.11-folds and 2.01-folds CLNM risks in SG and LG, respectively (SG: *OR*, 6.11, 95% *CI*, 3.16–11.81, *P* < 0.001; LG: *OR*, 2.01, 95% *CI*, 1.65–2.46, *P* < 0.001). Furthermore, significant publication biases were not founded in both subgroups.

## Discussion

In contrast to the better long-term survival prognosis of PTC, it has an unacceptable high incidence of CLNM, even in CN0 PTMC. The previous studies revealed the incidence of CLNM was about 18.3–50% in CN0 PTMC [23, 25]. Furthermore, CLNM was significantly associated with lateral lymph node metastases (LLNM) and a poorer prognosis of PTC [13, 26, 27]. Therefore, prophylactic CLND is widely performed in real-world surgical decisions, which brings clinical benefits in patients with intermediate- and high-risk PTC [28]. However, prophylactic CLND in pathological node-negative PTMC indeed brings unnecessary complication risks and economic burdens [29]. Exploring the preoperative and intraoperative clinicopathologic predictors of CLNM in CN0 PTMC may be a feasible method in the current situation without medical methods for accurate diagnosis of CLNM. The present studies documented that many clinicopathologic factors were significantly associated with CLNM in CN0 PTMC. For example, Gui, C et al. [15] previously reported a multivariate analysis in a cohort of 541 patients and demonstrated that male gender, age < 45 years, tumor size > 0.575 cm, multifocality, and gross ETE were independent risk factors for CLNM in CN0 PTMC. However, these studies have either been small sample studies or have confused definitions; the real and effective predictors of CLNM in CN0 PTMC remain unclear. The comprehensive systemic studies about predictors of CLNM in CN0 PTMC are limited. In this study, we performed a systematic review and meta-analysis to investigate the pooled effect of each candidate predictor.

To the best of our knowledge, this is the first meta-analysis to assess clinicopathologic risk factors for CLNM in CN0 PTMC. In this study, we found that male gender, multifocality, tumor size > 5 mm, and ETE were statistically significant clinicopathologic risk factors for CLNM in CN0 PTMC. Consistent with our finding, numerous studies identified the above clinicopathologic risk factors that were associated with lymph node metastases [26, 27]. Qu, H et al. [30] previously reported a systematic review and meta-analysis and demonstrated that male, multifocality, tumor size > 5 mm, capsular invasion, and ETE increased about 1.93-folds, 1.93-folds, 3.48-folds, 1.91-folds, and 2.42-folds of CLNM risk in PTC, respectively. Xue, S et al. [31] also reported age < 45, male, ETE, tumor size > 5 mm, and multifocality were significantly associated with increased a risk of LLNM in PTMC in their meta-analysis. Additionally, we found that male, multifocality, tumor size > 5mm, and ETE were increasing about 2.92-folds, 1.79-folds, 1.97-folds, and 1.84-folds CLNM risks in CN0 PTMC, respectively. Our results extended the effects of clinicopathologic risk factors on CLNM in CN0 PTMC and further confirmed that male gender, multifocality, tumor size > 5 mm, and ETE should be significant preoperative and intraoperative clinicopathologic predictors of CLNM in CN0 PTMC. Unfortunately, moderate heterogeneities were found in the final pooled analysis in both male and ETE. To explore the source of the heterogeneity, we applied sensitivity analysis and meta-regression. Fortunately, the sample size was identified as the source of the male gender. Then, the subgroup analysis of male was performed with a cutoff point of 200, which illustrated that male was increasing 6.11-folds and 2.01-folds CLNM risks in SG and LG, respectively. However, no significant variables were found in the meta-regression of ETE, mainly due to the insufficient quantity of the available studies. Moreover, publication biases occurred in the funnel plot and Egger’s test in male and ETE. The trim and filling analyses were applied, which finally conducted the calibrated value of pooled ORs. Fortunately, the results of the trim and filling analysis did not change the general direction of the conclusion, which demonstrated the calibrated value of ORs of male and ETE was 2.07 and 1.96, respectively. This study indicates that male gender, multifocality, tumor size > 5 mm, and ETE may be reliable predictors of CLNM. With careful evaluation of preoperative ultrasonography and intraoperative frozen pathology, prophylactic CLND should be considered in CN0 PTMC, when male gender, multifocality, tumor size > 5 mm, and ETE existed.

It is worth noting that many clinicopathologic factors were excluded from the pooled meta-analysis, which were significant risk factors in the individual included studies due to the insufficient amounts of eligible researches and the inconsistent definition of the same clinicopathologic factors. For example, age was investigated as a significant independent risk factor for CLNM in 5 included studies of our meta-analysis. However, the definitions of age contained 45 years and 50 years. According to existing studies, age was associated with aggressiveness of PTC, such as CLNM, LLNM, vascular invasion, and ETE [32, 33]. Niemann, A.C et al. [32] reported a multivariate analysis in a cohort of 632 patients and revealed that PTC patients aged < 25 years or aged > 75 years exhibited higher rates of aggressive histopathologic features compared to patients aged between 25 and 75 years. Oh HS [34] et al. also found that younger age was an independent predictor of large-volume CLNM in CN0 PTC. All of the above findings indicate that age may be a valuable candidate predictor for CLNM in CN0 PTMC. Further high-quality researches focusing on the effects of age with consistent definition are needed. Moreover, despite 10 studies that identified tumor size as an independent risk of CLNM in CN0 PTMC, only 5 studies were included in the final meta-analysis due to the inconsistent definitions of split points of tumor size, which contained 5 mm, 5.75 mm, 6 mm, and 7 mm. The insufficient quantity of the eligible studies may affect the accuracy of the OR value. Furthermore, bilateral, capsule involvement, tumor location, and HT were all excluded from the pooled meta-analysis, since not meeting the minimum criteria of 3 available studies. Tumor location and HT may be attractive predictors of CLNM in PTMC. Feng et al. and Zhang et al. revealed that superior localization of the tumor increased the risk of CLNM by 2.37-folds and 1.81-folds, respectively. The superior location of the tumor was not only associated with a high risk of CLNM but also might relate to skipping LLNM leaping over the central cervical compartment [35]. Our previous research had revealed that the effects of HT on CLNM in PTC depended on the different types of thyroid antibody status, suggesting that the cross talk between HT and PTC involves more complex mechanisms [36].

This meta-analysis has some limitations. First, the available studies included in the specific final meta-analysis were relatively insufficient due to the limited number of existing studies on risk factors of CLNM in CN0 PTMC, especially for the subgroup analysis of the ETE and tumor location. Second, heterogeneities and publication biases were found in some risk factors, which may affect the stability of the conclusion. Fortunately, the source of the heterogeneity was identified in male, and the subgroup analysis addressed the defect. Moreover, the results of the trim and filling analysis suggested that the general direction of the conclusion was unchanged. In addition, some potential significant predictors of CLNM in CN0 PTMC, such as age, were not included in the meta-analysis due to the inconsistent definition of split points. Nevertheless, the predictive values of male, multifocality, tumor size > 5 mm, and ETE for CLNM in CN0 PTMC were confirmed by our meta-analysis. Further updating of our meta-analysis with more eligible studies is still needed to identify more reliable predictors of CLNM in CN0 PTMC. These limitations remain to be explored in subsequent work.

## Conclusion

In summary, this systematic review and meta-analysis showed that male, multifocality, tumor size > 5 mm, and ETE were significantly associated with a higher risk of CLNM in CN0 PTMC. Specifically, compared to female, solitary, tumor size ≤ 5mm, and non-ETE PTMC, male, multifocality, tumor size > 5mm, and ETE increase 2.07-folds, 1.88-folds, 1.84-folds, and 1.96-folds risk of CLNM in CN0 PTMC, respectively. Male, multifocality, tumor size > 5 mm, and ETE may be reliable clinical predictors for CLNM in CN0 PTMC. Moreover, Prophylactic CLND should be considered in the surgical decision-making of CN0 PTMC patients, who are male, multifocal, with tumor size > 5 mm, and with ETE.

## Supplementary Information

**Additional file 1: Table S1.** PubMed search strategy. **Table S2.** Embase search strategy. **Table S3.** Cochrane Library search strategy. **Table S4.** Web of Science search strategy.

## Data Availability

The datasets used and/or analyzed during the current study are available from the corresponding author on reasonable request.

## Abbreviations

CI: Confidence intervals
CLND: Central lymph node dissection
CLNM: Central lymph node metastases
CN0: Clinical node-negative
ETE: Extrathyroidal extension
HT: Hashimoto’s thyroiditis
LG: Large sample group
LLNM: Lateral lymph node metastases
NOS: Newcastle-Ottawa scale
OR: Odds ratio
SG: Small sample group
PTC: Papillary thyroid carcinoma
PTMC: Papillary thyroid microcarcinoma

## References

[1] Miranda-Filho A, Lortet-Tieulent J, Bray F, Cao B, Franceschi S, Vaccarella S (2021). Thyroid cancer incidence trends by histology in 25 countries: a population-based study. Lancet Diabetes Endocrinol.

[2] Lim H, Devesa SS, Sosa JA, Check D, Kitahara CM (2017). Trends in thyroid cancer incidence and mortality in the United States, 1974-2013. JAMA.

[3] Li M, Dal Maso L, Vaccarella S (2020). Global trends in thyroid cancer incidence and the impact of overdiagnosis. Lancet Diabetes Endocrinol.

[4] Hedinger C, Williams ED, Sobin LH (1989). The WHO histological classification of thyroid tumors: a commentary on the second edition. Cancer Am Cancer Soc.

[5] Haugen BR, Alexander EK, Bible KC, Doherty GM, Mandel SJ, Nikiforov YE (2016). 2015 American Thyroid Association management guidelines for adult patients with thyroid nodules and differentiated thyroid cancer: the American Thyroid Association guidelines task force on thyroid nodules and differentiated thyroid cancer. Thyroid..

[6] Wrenn SM, Wang TS, Toumi A, Kiernan CM, Solórzano CC, Stephen AE (2021). Practice patterns for surgical management of low-risk papillary thyroid cancer from 2014 to 2019: A CESQIP analysis. Am J Surg.

[7] Patel KN, Yip L, Lubitz CC, Grubbs EG, Miller BS, Shen W (2020). Executive summary of the American Association of Endocrine Surgeons guidelines for the definitive surgical management of thyroid disease in adults. Ann Surg.

[8] Wang TS, Sosa JA (2018). Thyroid surgery for differentiated thyroid cancer - recent advances and future directions. Nat Rev Endocrinol.

[9] Zhou YL, Gao EL, Zhang W, Yang H, Guo GL, Zhang XH (2012). Factors predictive of papillary thyroid micro-carcinoma with bilateral involvement and central lymph node metastasis: a retrospective study. World J Surg Oncol.

[10] Yuan J, Li J, Chen X, Lin X, Du J, Zhao G (2017). Identification of risk factors of central lymph node metastasis and evaluation of the effect of prophylactic central neck dissection on migration of staging and risk stratification in patients with clinically node-negative papillary thyroid microcarcinoma. Bull Cancer.

[11] Zhang LY, Liu ZW, Liu YW, Gao WS, Zheng CJ (2015). Risk factors for nodal metastasis in cN0 papillary thyroid microcarcinoma. Asian Pac J Cancer Prev.

[12] Liu C, Liu H, Bian C, Yao XY, Wu Y, Chen SJ (2021). Preoperative risk factors and recommendations for surgical intervention in cN0 papillary thyroid microcarcinoma. Neoplasma..

[13] Feng JW, Pan H, Wang L, Ye J, Jiang Y, Qu Z (2019). Determine the optimal extent of thyroidectomy and lymphadenectomy for patients with papillary thyroid microcarcinoma. Front Endocrinol (Lausanne).

[14] Liu H, Li Y, Mao Y (2019). Local lymph node recurrence after central neck dissection in papillary thyroid cancers: a meta analysis. Eur Ann Otorhinolaryngol Head Neck Dis.

[15] Gui CY, Qiu SL, Peng ZH, Wang M (2018). Clinical and pathologic predictors of central lymph node metastasis in papillary thyroid microcarcinoma: a retrospective cohort study. J Endocrinol Investig.

[16] Kim BY, Jung CH, Kim JW, Lee SW, Kim CH, Kang SK (2012). Impact of clinicopathologic factors on subclinical central lymph node metastasis in papillary thyroid microcarcinoma. Yonsei Med J.

[17] Park JP, Roh JL, Lee JH, Baek JH, Gong G, Cho KJ (2014). Risk factors for central neck lymph node metastasis of clinically noninvasive, node-negative papillary thyroid microcarcinoma. Am J Surg.

[18] Xu D, Lv X, Wang S, Dai W (2014). Risk factors for predicting central lymph node metastasis in papillary thyroid microcarcinoma. Int J Clin Exp Pathol.

[19] So YK, Son YI, Hong SD, Seo MY, Baek CH, Jeong HS (2010). Subclinical lymph node metastasis in papillary thyroid microcarcinoma: a study of 551 resections. Surgery..

[20] Caliskan M, Park JH, Jeong JS, Lee CR, Park SK, Kang SW (2012). Role of prophylactic ipsilateral central compartment lymph node dissection in papillary thyroid microcarcinoma. Endocr J.

[21] Zhang Q, Wang Z, Meng X, Duh Q, Chen G (2019). Predictors for central lymph node metastases in CN0 papillary thyroid microcarcinoma (mPTC): a retrospective analysis of 1304 cases. Asian J Surg.

[22] Feng JW, Ye J, Wu WX, Qu Z, Qin AC, Jiang Y (2020). Management of cN0 papillary thyroid microcarcinoma patients according to risk-scoring model for central lymph node metastasis and predictors of recurrence. J Endocrinol Investig.

[23] Zhang C, Li BJ, Liu Z, Wang LL, Cheng W (2020). Predicting the factors associated with central lymph node metastasis in clinical node-negative (cN0) papillary thyroid microcarcinoma. Eur Arch Otorhinolaryngol.

[24] Liberati A, Altman DG, Tetzlaff J, Mulrow C, Gøtzsche PC, Ioannidis JP (2009). The PRISMA statement for reporting systematic reviews and meta-analyses of studies that evaluate health care interventions: explanation and elaboration. PLoS Med.

[25] Kim BY, Choi N, Kim SW, Jeong HS, Chung MK, Son YI (2020). Randomized trial of prophylactic ipsilateral central lymph node dissection in patients with clinically node negative papillary thyroid microcarcinoma. Eur Arch Otorhinolaryngol.

[26] Zheng X, Peng C, Gao M, Zhi J, Hou X, Zhao J (2019). Risk factors for cervical lymph node metastasis in papillary thyroid microcarcinoma: a study of 1,587 patients. Cancer Biol Med.

[27] Siddiqui S, White MG, Antic T, Grogan RH, Angelos P, Kaplan EL (2016). Clinical and pathologic predictors of lymph node metastasis and recurrence in papillary thyroid microcarcinoma. Thyroid..

[28] Medas F, Canu GL, Cappellacci F, Anedda G, Conzo G, Erdas E (2020). Prophylactic central lymph node dissection improves disease-free survival in patients with intermediate and high risk differentiated thyroid carcinoma: a retrospective analysis on 399 patients. Cancers (Basel).

[29] Giordano D, Valcavi R, Thompson GB, Pedroni C, Renna L, Gradoni P (2012). Complications of central neck dissection in patients with papillary thyroid carcinoma: results of a study on 1087 patients and review of the literature. Thyroid..

[30] Qu H, Sun G, Liu Y, He Q (2015). Clinical risk factors for central lymph node metastasis in papillary thyroid carcinoma: a systematic review and meta-analysis. Clin Endocrinol.

[31] Xue S, Han Z, Lu Q, Wang P, Chen G (2020). Clinical and ultrasonic risk factors for lateral lymph node metastasis in papillary thyroid microcarcinoma: a systematic review and meta-analysis. Front Oncol.

[32] Niemann AC, Reid AT, Smith J, Hammond J, DeBolle SA, Wei I (2017). Association of patient age with high-risk pathologic features in papillary thyroid cancer. J Surg Res.

[33] Ito Y, Miyauchi A, Kihara M, Higashiyama T, Kobayashi K, Miya A (2014). Prognostic significance of young age in papillary thyroid carcinoma: analysis of 5,733 patients with 150 months’ median follow-up. Endocr J.

[34] Oh HS, Park S, Kim M, Kwon H, Song E, Sung TY (2017). Young age and male sex are predictors of large-volume central neck lymph node metastasis in clinical N0 papillary thyroid microcarcinomas. Thyroid..

[35] Wang W, Yang Z, Ouyang Q (2020). A nomogram to predict skip metastasis in papillary thyroid cancer. World J Surg Oncol.

[36] Wen X, Wang B, Jin Q, Zhang W, Qiu M (2019). Thyroid antibody status is associated with central lymph node metastases in papillary thyroid carcinoma patients with Hashimoto’s thyroiditis. Ann Surg Oncol.

