# Supplementary material for: Clinicopathologic predictors of central lymph node metastases in clinical node-negative papillary thyroid microcarcinoma: a systematic review and meta-analysis
Source: World J Surg Oncol. 2022 Apr 1;20:106. doi: 10.1186/s12957-022-02573-7 (PMC8976349; doi:10.1186/s12957-022-02573-7)
Supplement: Supplementary file 1 — Additional file 1: Table S1. PubMed search strategy. Table S2. Embase search strategy. Table S3. Cochrane Library search strategy. Table S4. Web of Science search strategy. [file 12957_2022_2573_MOESM1_ESM.docx]

**The Detailed Search Strategy for Each of Database**

A literature search was conducted from PubMed, Embase, Cochrane Library and Web of Science for articles published until February 11, 2022. A combination of the following terms: ‘‘Papillary Thyroid Microcarcinoma’’, ‘‘Lymphatic Metastasis’’, ‘‘Risk factors’’, and ‘‘Case-Control Studies’’ were applied according to the “PICOS” principle. The subject words and related random words were listed as follow:

**Participants**. The subject word was “Papillary Thyroid Microcarcinoma”, and the random words were “Papillary Thyroid Microcarcinoma*”, “Thyroid Papillary Microcarcinoma*”, and “PTMC”.

**Interventions and comparisons (exposure factors).** The subject word was “Lymphatic Metastasis”, and the random words were “Lymphatic Metastases”, “Lymph Node Metastasis”, “Lymph Node Metastases”, and “Metastasis, Lymph Node”.

**Outcomes.** The subject word was “Risk factors”, and the random words were “Factor, Risk”, “Factors, Risk”, and “Risk Factor”.

**Studies.** The subject word was “Case-Control Studies” and “Cohort Studies”. The random words were “Case-Control Study”, “Studies, Case-Control”, “Study, Case-Control”, “Case-Comparison Studies”, “Case Comparison Studies”, “Case-Comparison Study”, “Studies, Case-Comparison”, “Study, Case-Comparison”, “Case Control Studies”, “Case Control Study”, “Studies, Case Control”, “Study, Case Control”, “Cohort Study”, “Studies, Cohort”, and “Study, Cohort”.

The detailed search strategies of PubMed, Embase, Cochrane Library and Web of Science were presented as followed (Table S1-4).

| **Table S1.** PubMed search strategy | |
| --- | --- |
| #1 | "Papillary Thyroid Microcarcinoma"[Supplementary Concept] |
| #2 | " Papillary Thyroid Microcarcinoma*"[Title/Abstract] OR " Thyroid Papillary Microcarcinoma*"[Title/Abstract] OR "PTMC"[Title/Abstract] |
| #3 | #1 OR #2 |
| #4 | "Lymphatic Metastasis"[MeSH Terms] |
| #5 | "Lymphatic Metastases"[Title/Abstract] OR "Lymph Node Metastasis"[Title/Abstract] OR "Lymph Node Metastases"[Title/Abstract] OR " Metastasis, Lymph Node "[Title/Abstract] |
| #6 | #4 OR #5 |
| #7 | "Risk Factors"[MeSH Terms] |
| #8 | "Factor, Risk"[Title/Abstract] OR "Factors, Risk"[Title/Abstract] OR "Risk Factor"[Title/Abstract] |
| #9 | #7 OR #8 |
| #10 | "Case-Control Studies"[MeSH Terms] |
| #11 | "Cohort Studies"[MeSH Terms] |
| #12 | " Case-Control Study"[Title/Abstract] OR "Studies, Case-control"[Title/Abstract] OR "Study, case-control"[Title/Abstract] OR "Case-comparison Studies"[Title/Abstract] OR "Case Comparison Studies"[Title/Abstract] OR "Case-comparison Study"[Title/Abstract] OR "Studies, Case-Comparison"[Title/Abstract] OR "Study, Case-Comparison"[Title/Abstract] OR "Case Control Studies"[Title/Abstract] OR "Case Control Study"[Title/Abstract] OR "Studies, Case Control"[Title/Abstract] OR "Study, Case Control"[Title/Abstract] OR "Cohort Study"[Title/Abstract] OR "Studies, Cohort"[Title/Abstract] OR "Study, Cohort"[Title/Abstract] |
| #13 | #10 OR #11 OR #12 |
| #14 | #3 AND #6 AND #9 AND #13 |

| **Table S2.** Embase search strategy | |
| --- | --- |
| #1 | Papillary Thyroid Microcarcinoma |
| #2 | 'Papillary Thyroid Microcarcinoma* ':ab,ti OR 'PTMC':ab,ti OR 'Thyroid Papillary Microcarcinoma*':ab,ti |
| #3 | #1 OR #2 |
| #4 | Lymphatic Metastasis |
| #5 | 'Lymphatic Metastases':ab,ti OR 'Lymph Node Metastasis':ab,ti OR 'Lymph Node Metastases':ab,ti OR 'Metastasis, Lymph Node':ab,ti |
| #6 | #4 OR #5 |
| #7 | Risk Factors |
| #8 | 'Factor, Risk':ab,ti OR 'Factors, Risk':ab,ti OR 'Risk Factor':ab,ti |
| #9 | #7 OR #8 |
| #10 | Case-Control Studies |
| #11 | Cohort Studies |
| #12 | 'Case-Control Study':ab,ti OR 'Studies, Case-Control':ab,ti OR 'Study, Case-Control':ab,ti OR 'Case-Comparison Studies':ab,ti OR 'Case Comparison Studies':ab,ti OR 'Case-Comparison Study':ab,ti OR 'Studies, Case-Comparison':ab,ti OR 'Study, Case-Comparison':ab,ti OR 'Case Control Studies':ab,ti OR 'Case Control Study':ab,ti OR 'Studies, Case Control':ab,ti OR 'Study, Case Control':ab,ti OR 'Cohort Study':ab,ti OR 'Studies, Cohort':ab,ti OR 'Study, Cohort':ab,ti |
| #13 | #10 OR #11 OR #12 |
| #14 | #3 AND #6 AND #9 AND #13 |

| **Table S3.** Cochrane Library search strategy | |
| --- | --- |
| #1 | Papillary Thyroid Microcarcinoma |
| #2 | (Papillary Thyroid Microcarcinoma* ):ab,ti,kw OR (PTMC):ab,ti,kw OR (Thyroid Papillary Microcarcinoma*):ab,ti,kw |
| #3 | #1 OR #2 |
| #4 | Lymphatic Metastasis |
| #5 | (Lymphatic Metastases):ab,ti,kw OR (Lymph Node Metastasis):ab,ti,kw OR (Lymph Node Metastases):ab,ti,kw OR (Metastasis, Lymph Node):ab,ti,kw |
| #6 | #4 OR #5 |
| #7 | Risk Factors |
| #8 | (Factor, Risk):ab,ti,kw OR (Factors, Risk):ab,ti,kw OR (Risk Factor):ab,ti,kw |
| #9 | #7 OR #8 |
| #10 | Case-Control Studies |
| #11 | Cohort Studies |
| #12 | (Case-Control Study):ab,ti,kw OR (Studies, Case-Control):ab,ti,kw OR (Study, Case-Control):ab,ti,kw OR (Case-Comparison Studies):ab,ti,kw OR (Case Comparison Studies):ab,ti,kw OR (Case-Comparison Study):ab,ti,kw OR (Studies, Case-Comparison):ab,ti,kw OR (Study, Case-Comparison):ab,ti,kw OR (Case Control Studies):ab,ti,kw OR (Case Control Study):ab,ti,kw OR (Studies, Case Control):ab,ti,kw OR (Study, Case Control):ab,ti,kw OR (Cohort Study):ab,ti,kw OR (Studies, Cohort):ab,ti,kw OR (Study, Cohort):ab,ti,kw |
| #13 | #10 OR #11 OR #12 |
| #14 | #3 AND #6 AND #9 AND #13 |

| **Table S4.** Web of Science search strategy | |
| --- | --- |
| #1 | TS=(Papillary Thyroid Microcarcinoma* OR PTMC OR Thyroid Papillary Microcarcinoma*) |
| #2 | TS=(Lymphatic Metastasis OR Lymphatic Metastases OR Lymph Node Metastasis OR Lymph Node Metastases OR Metastasis, Lymph Node) |
| #3 | TS=(Risk Factors OR Factor, Risk OR Factors, Risk OR Risk Factor) |
| #4 | TS=(Case-Control Studies OR Cohort Studies OR Case-Control Study OR Studies, Case-Control OR Study, Case-Control OR Case-Comparison Studies OR Case Comparison Studies OR Case-Comparison Study OR Studies, Case-Comparison OR Study, Case-Comparison OR Case Control Studies OR Case Control Study OR Studies, Case Control OR Study, Case Control OR Cohort Study OR Studies, Cohort OR Study, Cohort ) |
| #5 | #1 AND #2 AND #3 AND #4 |
